# Persistently high incidence rates of childhood acute leukemias from 2010 to 2017 in Mexico City: A population study from the MIGICCL

**DOI:** 10.3389/fpubh.2022.918921

**Published:** 2022-09-14

**Authors:** Janet Flores-Lujano, David Aldebarán Duarte-Rodríguez, Elva Jiménez-Hernández, Jorge Alfonso Martín-Trejo, Aldo Allende-López, José Gabriel Peñaloza-González, María Luisa Pérez-Saldivar, Aurora Medina-Sanson, José Refugio Torres-Nava, Karina Anastacia Solís-Labastida, Luz Victoria Flores-Villegas, Rosa Martha Espinosa-Elizondo, Raquel Amador-Sánchez, Martha Margarita Velázquez-Aviña, Laura Elizabeth Merino-Pasaye, Nora Nancy Núñez-Villegas, Ana Itamar González-Ávila, María de los Ángeles del Campo-Martínez, Martha Alvarado-Ibarra, Vilma Carolina Bekker-Méndez, Rocío Cárdenas-Cardos, Silvia Jiménez-Morales, Roberto Rivera-Luna, Haydee Rosas-Vargas, Norma C. López-Santiago, Angélica Rangel-López, Alfredo Hidalgo-Miranda, Elizabeth Vega, Minerva Mata-Rocha, Omar Alejandro Sepúlveda-Robles, José Arellano-Galindo, Juan Carlos Núñez-Enríquez, Juan Manuel Mejía-Aranguré

**Affiliations:** ^1^Unidad de Investigación Médica en Epidemiología Clínica, Unidad Médica de Alta Especialidad, Hospital de Pediatría “Dr. Silvestre Frenk Freund, ” Centro Médico Nacional Siglo XXI, Instituto Mexicano del Seguro Social (IMSS), Mexico City, Mexico; ^2^Servicio de Hematología Pediátrica, Centro Médico Nacional “La Raza, ” Hospital General “Gaudencio González Garza, ” Instituto Mexicano del Seguro Social (IMSS), Mexico City, Mexico; ^3^Servicio de Oncología, Hospital Pediátrico de Moctezuma, Secretaría de Salud de la Ciudad de México (SSCDMX), Mexico City, Mexico; ^4^Servicio de Hematología Pediátrica, Unidad Médica de Alta Especialidad, Hospital de Pediatría “Dr. Silvestre Frenk Freund, ” Centro Médico Nacional “Siglo XXI, ” Instituto Mexicano del Seguro Social (IMSS), Mexico City, Mexico; ^5^Servicio de OncoPediatría, Hospital Juárez de México, Secretaría de Salud (SS), Mexico City, Mexico; ^6^Departamento de HematoOncología, Hospital Infantil de México Federico Gómez, Secretaría de Salud (SS), Mexico City, Mexico; ^7^Servicio de Hematología Pediátrica, Centro Médico Nacional “20 de Noviembre, ” Instituto de Seguridad y Servicios Sociales de los Trabajadores del Estado (ISSSTE), Mexico City, Mexico; ^8^Servicio de Hematología Pediátrica, Hospital General de México, Secretaría de Salud (SS), Mexico City, Mexico; ^9^Servicio de Hematología Pediátrica, Hospital General Regional 1 “Dr. Carlos McGregor Sánchez Navarro, ” Instituto Mexicano del Seguro Social (IMSS), Mexico City, Mexico; ^10^Hospital de Infectología “Dr. Daniel Méndez Hernández, ” “La Raza, ” Instituto Mexicano del Seguro Social (IMSS), Unidad de Investigación Médica en Inmunología e Infectología, Mexico City, Mexico; ^11^Servicio de Oncología Pediátrica, Instituto Nacional de Pediatría, Secretaría de Salud (SS), Mexico City, Mexico; ^12^Laboratorio de Genómica del Cáncer, Instituto Nacional de Medicina Genómica (INMEGEN), Mexico City, Mexico; ^13^Unidad de Investigación Médica en Genética Humana, Unidad Médica de Alta Especialidad, Hospital de Pediatría “Dr. Silvestre Frenk Freund, ” Centro Médico Nacional Siglo XXI, Instituto Mexicano del Seguro Social (IMSS), Mexico City, Mexico; ^14^Servicio de Hematología Pediátrica, Instituto Nacional de Pediatría, Secretaría de Salud (SS), Mexico City, Mexico; ^15^Coordinación de Investigación en Salud, Unidad Habilitada de Apoyo al Predictamen, Centro Médico Siglo XXI, Instituto Mexicano del Seguro Social, Mexico City, Mexico; ^16^Instituto de Ciencias de la Atmósfera y Cambio Climático, Universidad Nacional Autónoma de México (UNAM), Mexico City, Mexico; ^17^Unidad de Investigación en Enfermedades Infecciosas, Laboratorio de Virología Clínica y Experimental, Hospital Infantil de México Federico Gómez, Secretaría de Salud (SS), Mexico City, Mexico; ^18^Facultad de Medicina, Universidad Nacional Autónoma de México (UNAM), Mexico City, Mexico

**Keywords:** incidence, childhood, acute leukemia, acute lymphoblastic leukemia, acute myeloblastic leukemia, Mexican population, epidemiology

## Abstract

**Introduction:**

Over the years, the Hispanic population living in the United States has consistently shown high incidence rates of childhood acute leukemias (AL). Similarly, high AL incidence was previously observed in Mexico City (MC). Here, we estimated the AL incidence rates among children under 15 years of age in MC during the period 2010–2017.

**Methods:**

The Mexican Interinstitutional Group for the Identification of the Causes of Childhood Leukemia conducted a study gathering clinical and epidemiological information regarding children newly diagnosed with AL at public health institutions of MC. Crude age incidence rates (cAIR) were obtained. Age-standardized incidence rates worldwide (ASIRw) and by municipalities (ASIRm) were calculated by the direct and indirect methods, respectively. These were reported per million population <15 years of age; stratified by age group, sex, AL subtypes, immunophenotype and gene rearrangements.

**Results:**

A total of 903 AL cases were registered. The ASIRw was 63.3 (cases per million) for AL, 53.1 for acute lymphoblastic leukemia (ALL), and 9.4 for acute myeloblastic leukemia. The highest cAIR for AL was observed in the age group between 1 and 4 years (male: 102.34 and female: 82.73). By immunophenotype, the ASIRw was 47.3 for B-cell and 3.7 for T-cell. The incidence did not show any significant trends during the study period. The ASIRm for ALL were 68.6, 66.6 and 62.8 at Iztacalco, Venustiano Carranza and Benito Juárez, respectively, whereas, other municipalities exhibited null values mainly for AML.

**Conclusion:**

The ASIRw for childhood AL in MC is among the highest reported worldwide. We observed spatial heterogeneity of rates by municipalities. The elevated AL incidence observed in Mexican children may be explained by a combination of genetic background and exposure to environmental risk factors.

## Introduction

Acute leukemias (AL) are the most common childhood cancer worldwide (1), representing over one-third of all childhood cancer types (2). Populations with Hispanic (Latin American) ancestry living in the United States have exhibited high AL incidences, as also documented previously in Mexican children (3–6). Among Hispanics, the incidence of childhood AL is 55.0 cases per million children under 15 years of age. These rates are higher compared to those reported for other ethnic groups, such as White, Asian-Pacific, and African-American (7). In Mexico City (MC), the incidence rates of AL are among the highest in the world (3, 8–10) (Supplementary Table 1). Recent studies have reported an increasing trend in AL incidence among children (1.1 and 2.3% annually), particularly, in populations of Hispanic ancestry (7, 11–13); however, this tendency has not been consistently observed, neither broadly (14) nor particularly in MC (10).

Acute lymphoblastic leukemia (ALL) is the most frequent leukemia subtype in Mexican children where the B-cell ALL, also known as B-ALL, occurs with frequencies of 76.1–81.4%, followed by the T-cell subtype (12.4–23.6%) (3, 15). These immunophenotypes display frequencies of 87 and 13% in the Hispanic population, and 85 and 15% worldwide, respectively (16–23). Additionally, the prevalence of the most common gene rearrangements in Mexican population is as follows: *ETV6-RUNX1* (7.4–13.5%), *TCF3-PBX1* (7.1–11.5%), *BCR-ABL1* (1.8–2.7%), and *MLL* rearrangements (1.4–8.7%) (24–26). These frequencies are similar to those found in other Hispanic populations (24, 25, 27–29), and those reported worldwide (13, 20, 23–25, 27–41). One important and recurrent finding in the Mexican population with childhood ALL is the low frequency of *ETV6-RUNX1*, a gene rearrangement associated with a good prognosis of the disease (42). Internationally, the prevailing assumption was that these gene rearrangements were the most important ones, which played an important role in the clinic (diagnosis, risk stratification and chemotherapy treatment). However, new genetic variants have been identified that provide additional information not only on the implications for treatment and relapse of this disease, but also on the ages at which they are most commonly observed (43).

MC not only exhibits among the highest AL incidence rates in the world, but it has also shown variable incidence trends over time, suggesting a potential role of non-genetic factors in disease development (44), such as an effect of the diversity of environmental exposure across municipalities (45). Several studies support this possibility, exhibiting differences in exposure among areas or municipalities—for example, to air pollution (46–49), metalsand chemicals of industrial, agriculture, commercial, and vehicular origin (50, 51), and to different near-surface air temperatures. This last variable has become of great epidemiological interest (52–56) since it is related to the atmospheric distribution of pollutants, and shows great variability between municipalities and areas of MC (57).

MC is composed by 16 municipalities, previously defined such as boroughs by political reasons (until 2018). No changes in delineation of these have occurred in the past 50 years (58, 59). The municipalities of Cuauhtémoc, Benito Juárez, Miguel Hidalgo, Azcapotzalco are in the central and northern territory; and to the south and southwest are the municipalities of Álvaro Obregón and Coyoacán. These have an abundance of fresh water, greater urban development, business, and service areas and have a medium population density. Also, these zones have greater railroad connections, which favored greater industrial development.

On the other hand, located to the east of MC are the municipalities of Iztapalapa, Iztacalco, and Venustiano Carranza; and to the northeast, the Gustavo A. Madero. These municipalities are densely populated and considered as a low socioeconomic status areas. The east of the city has a considerable extension of plain land which led to the construction of the airport of MC.

In the south of MC, the municipalities of La Magdalena Contreras, Cuajimalpa, Tlalpan, Xochimilco and Tláhuac are located. These have been the last to be urbanized, and even rural and agricultural activities still subsist. Currently, the municipality of Milpa Alta, located in the southeast, is almost entirely rural (60–62).

Moreover, MC possesses a bright land surface and a peculiar weather cycle and is in a high-altitude and semi-enclosed basin surrounded by mountains with unique planetary boundary layer dynamics (46). In addition, a spatial variability of mortality rates by municipalities has been reported in MC (63). Therefore, it is important to perform a constant monitoring and evaluation of AL trends of incidence rates by municipalities in MC.

In the present study, we aimed to assess the AL incidence rate and trends (age, sex, immunophenotype, and gene rearrangement) during the period 2010–2017, MC residents of 0–14 years of age, according to age groups and municipalities.

## Materials and methods

AL cases were identified using the population-based registry of the Mexican Interinstitutional Group for the Identification of the Causes of Childhood Leukemia (MIGICCL). Since 2006, the MIGICCL has collected information from public health institutions in MC: *Instituto Mexicano del Seguro Social* (IMSS) (three hospitals)*, Instituto de Seguridad y Servicios Sociales de los Trabajadores del Estado* (ISSSTE) (two hospitals)*, Secretar*í*a de Salud* (SS) (four hospitals), and *Secretar*í*a de Salud de la Ciudad de México* (SS CDMX) (one hospital). These 10 hospitals provide care for an estimated 97.5% of children with leukemia who reside in MC (64).

### Validity of the methods

Case registration required that trained personnel were assigned to each participating hospital to identify incident cases of leukemia through reviews of clinical charts. The parents of each child were required to sign an informed consent form if they agreed to participate in an in-person interview.

The interview was carried out using a questionnaire previously standardized and adapted from the questionnaire module of the National Cancer Institute. General information obtained included the child's sex, age at the time of diagnosis (in years), and municipality of residence. Additionally, the clinical chart of the child and an official document (photo-voting card) provided by the parents at the time of the interview were reviewed, when possible, to verify the accuracy of the information provided.

AL diagnosis was established based on clinical features, and bone marrow aspirate findings, including cell morphology, immunophenotype, and genetics, as defined in 2008 by the World Health Organization (WHO) for the classification of lymphoid neoplasms.

The MIGICCL registered all newly diagnosed AL cases from January 2010 to December 2017. Only cases with residence in MC were included.

The leukemia subtypes were defined according to ICD-O-2/ICD-O-3, forming the following three groups: ALL (9820, 9821, 9828), AML (9840, 9860, 9861, 9866, 9867, 9872, 9873, 9874, 9891, 9910), and other leukemias (OL) (9863, 9868, 9875, 880) (65). Furthermore, the immunophenotypes were classified according to WHO criteria: B-cell, T-cell, and AL of ambiguous lineage (ALAL) (66).

Gene rearrangements were detected by experts in two IMSS laboratories. The four most common gene rearrangements with clinical implications (*ETV6-RUNX1, TCF3-PBX1, BCR-ABL1*, and *MLL* rearrangements) were identified by reverse transcription polymerase chain reaction (RT-PCR) using a bone marrow sample that was collected by pediatric hematologists or oncologists from each patient at the time of diagnosis confirmation (67). The assessment of these gene rearrangements in the IMSS' laboratories began in 2010 through financial support of research studies and they were not routinely performed in most of the public institutions of MC.

#### Populations

The population base of children under 15 years old from each municipality was obtained through information from the 2000 and 2010 censuses of the National Institute of Statistics and Geography (INEGI) (68–71). The exponential projection method was used to estimate the population of children from 2011 to 2017 (72, 73).

#### Incidence rate estimation

The crude incidence rate was reported by age-specific groups (cAIR). This was calculated by dividing the number of new AL cases from each year (2010–2017) by the population of children from each municipality of MC during the same period. Afterwards, the rates were determined for overall AL and subtypes (ALL, AML and OL). In addition, the rates were calculated according to the immunophenotype classification (B-cell, T-cell, and ALAL) (66), gene rearrangement groups (*TCF3-PBX1, ETV6-RUNX1, MLL-AFF1, BCR-ABL1*, and other) and by the child's sex. These rates were reported per million children under 15 years of age.

Standardized incidence rates were calculated using the direct and indirect methods, they were also reported per million children under 15 years of age. The direct method compared age-standardized incidence rates of AL worldwide (ASIRw), with the reference being the world population as proposed by Segi (74), and modified by Doll et al. (75). Analyses were performed according to age groups (0–4, 5–9, and 10–14 years old), as well as by sex, AL subtype, immunophenotype, and gene rearrangements. The indirect method calculated the age-standardized incidence rates by municipalities (ASIRm), with the reference being population data reported in the 2010 census from INEGI for MC. Analyses were performed according to age groups (<1, 1–4, 5–9, and 10–14 years old), as well as by sex, AL subtype, immunophenotype, and gene rearrangements. We used this approach because some municipalities and age groups showed a lower or even null frequency of some AL subtypes. To understand the spatial distribution of incidence in each municipality, the ASIRm results were mapped using QGIS 3.14 software (Open Source Geospatial Foundation, Beaverton, Oregon, USA, QGIS, RRID:SCR_018507).

We also evaluated whether the incidence rates showed a temporal trend, among different AL subgroups, by estimating the average annual percent change (AAPC), using the Joinpoint Regression Program, Version 4.8.0.1 (National Cancer Institute, Bethesda, Maryland, USA, Joinpoint, RRID:SCR_018129). This program does not analyze years with incidence values of zero. To overcome this limitation, we used a correction strategy that has been applied in other studies (76, 77), in which an imputed incidence rate was generated by arbitrarily adding a half-case within the age group with the largest child population to the calculation. However, the AAPC could not be calculated when the correction required imputed data for more than 3 years.

## Results

**Figure 1:**
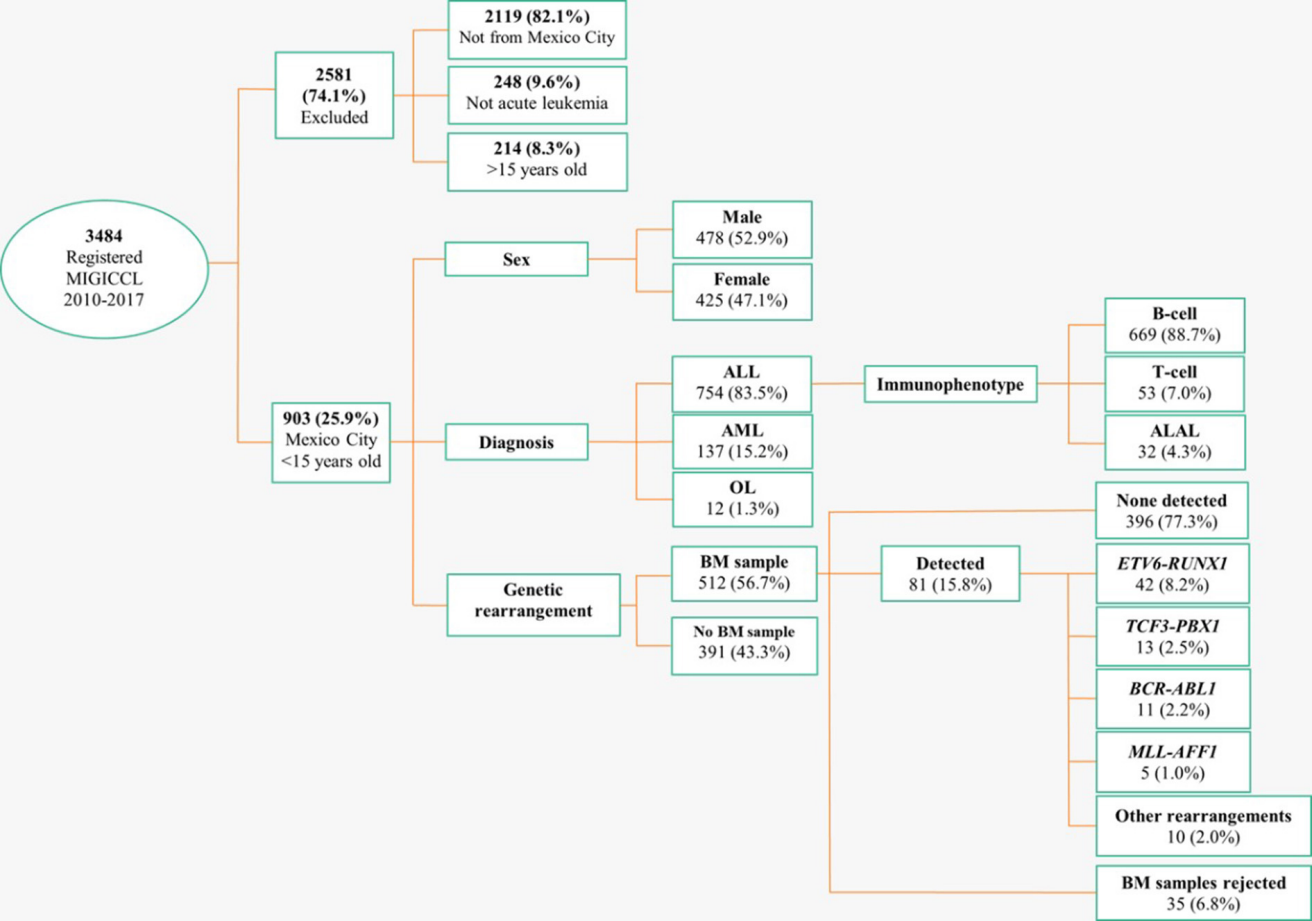
Flow chart of the selection process. The incidence rate of AL among children of MC during the period 2010–2017 based on the MIGICCL registry. MIGICCL, Mexican Interinstitutional Group for Identification of the Causes of Childhood Leukemia; ALL, acute lymphoblastic leukemia; AML, acute myeloblastic leukemia; OL, other leukemias; BM, bone marrow; ALAL, AL of ambiguous lineage.

A total of 3,484 incident cases were registered in the MIGICCL database during the period from 2010 to 2017, among which 903 (25.9%) patients were residents of MC (Figure 1). Of the included cases, 478 patients were male (52.9%) and 425 were female (47.1%). The diagnosis was ALL in 754 (83.5%) patients, AML in 137 (15.2%), and OL in 12 (1.3%). The most common subtype was B-cell (*n* = 669, 88.7%), followed by T-cell (7.0%) and ALAL (4.3%). Gene rearrangement data were available for 512 (56.7%) patients. Of these patients, 396 (77.4%) were negative for the four most common fusion genes. *ETV6-RUNX1* was detected in 42 (8.2%) cases, and *TCF3-PBX1* in 13 (2.5%) (Figure 1).

### AL incidence rates in MC

The cAIR for all AL was 61.1 cases per million. By AL subtype, the cAIR was 51.0 for ALL, 9.3 for AML, and 0.8 for OL. The ASIRw was 63.3 cases per million for all AL, 53.1 for ALL, 9.4 for AML, and 0.8 for OL (Table 1).

**Table 1:** The incidence rate of AL among children in MC, based on the MIGCCL registry during the period 2010–2017.

**AL**	**Sex**	**Age-specific rate**								**0–14 y**		**ASIRw**	**APC (95% CI)**
		<**1**		**1–4**		**5–9**		**10–14**					
		***n***	**cAIR**	***n***	**cAIR**	***n***	**cAIR**	***n***	**cAIR**	***n***	**cAIR**		
All-AL	Male	6	14.06	200	102.34	130	51.29	142	55.08	478	63.78	66.03	−4.2 (−11.8, 4)
	Female	20	48.88	156	82.73	138	55.46	111	44.34	425	58.35	60.45	
	Total	26	31.11	356	92.71	268	53.34	253	49.79	903	61.11	63.28	
**Subtype**													
ALL	Male	2	4.69	172	88.01	112	43.85	109	42.28	395	52.59	54.71	−3.3 (−7.6, 1.3)
	Female	16	39.1	137	72.65	119	48.17	87	34.75	359	49.4	51.44	
	Total	18	21.54	309	80.47	231	45.97	196	38.57	754	51.02	53.1	
AML	Male	3	7.03	25	12.79	17	7.05	29	11.25	74	9.99	10.09	0.7 (−10.2, 12.9)
	Female	3	7.33	19	10.08	17	6.48	24	9.59	63	8.53	8.58	
	Total	6	7.18	44	11.46	34	6.77	53	10.43	137	9.27	9.35	
Other	Male	1	2.34	3	1.54	1	0.39	4	1.55	9	1.2	1.23	–
	Female	1	2.44	0	0	2	0.81	0	0	3	0.41	0.43	
	Total	2	2.39	3	0.78	3	0.6	4	0.79	12	0.81	0.84	
**Immunophenotype**													
B-cell	Male	2	4.69	151	77.26	95	37.19	88	34.14	336	44.74	46.78	−2.6 (−7.8, 2.8)
	Female	15	36.66	128	67.88	109	44.12	81	32.35	333	45.83	47.75	
	Total	17	20.34	279	72.65	204	40.6	169	33.26	669	45.27	47.26	
T-cell	Male	0	0	14	7.16	11	4.31	16	6.98	41	5.73	5.69	−4.7 (−12.3, 3.6)
	Female	0	0	5	2.65	4	1.62	3	0.4	12	1.38	1.48	
	Total	0	0	19	4.95	15	2.99	19	3.74	53	3.59	3.62	
ALAL	Male	0	0	7	3.58	6	2.35	5	2.33	18	2.53	2.57	−19.3 (−38.4, 5.7)
	Female	1	2.44	4	2.12	6	2.43	3	0.8	14	1.79	1.86	
	Total	1	1.2	11	2.86	12	2.39	8	1.57	32	2.17	2.22	
**Gene rearrangement**													
TCF3-PBX1	Male	0	0	4	2.05	0	0	2	0.78	6	0.8	0.88	−8.1 (−20.8, 6.6)
	Female	0	0	4	2.12	1	0.4	2	0.8	7	0.96	1.04	
	Total	0	0	8	2.08	1	0.2	4	0.79	13	0.88	0.96	
ETV6-RUNX1	Male	0	0	8	4.09	5	1.96	2	0.78	15	2	2.16	10.7 (−3.1, 26.6)
	Female	0	0	10	5.3	12	4.86	5	2	27	3.72	3.83	
	Total	0	0	18	4.69	17	3.38	7	1.38	42	2.84	2.98	
MLL-AFF1	Male	0	0	1	0.51	1	0.39	0	0	2	0.27	0.29	–
	Female	1	2.44	1	0.53	1	0.4	0	0	3	0.41	0.47	
	Total	1	1.2	2	0.52	2	0.4	0	0	5	0.34	0.38	
BCR-ABL1	Male	0	0	1	0.51	1	0.39	2	0.78	4	0.53	0.51	–
	Female	0	0	1	0.53	4	1.62	2	0.8	7	0.96	0.92	
	Total	0	0	2	0.52	5	1	4	0.79	11	0.74	0.72	
Other rearrangement	Male	0	0	2	1.02	1	0.39	0	0	3	0.4	0.45	–
	Female	0	0	2	1.06	2	0.81	3	1.2	7	0.96	0.95	
	Total	0	0	4	1.04	3	0.6	3	0.59	10	0.68	0.7	

MIGCCL, Mexican Interinstitutional Group for the Identification of the Causes of Childhood Leukemia; ALAL, AL of ambiguous lineage; APC, annual percent change; cAIR, crude age incidence rates; ASIRw, age-standardized incidence rates (using Segi World Standard Population) per million children.

### AL incidence rates in MC by municipalities

**Figure 2:**
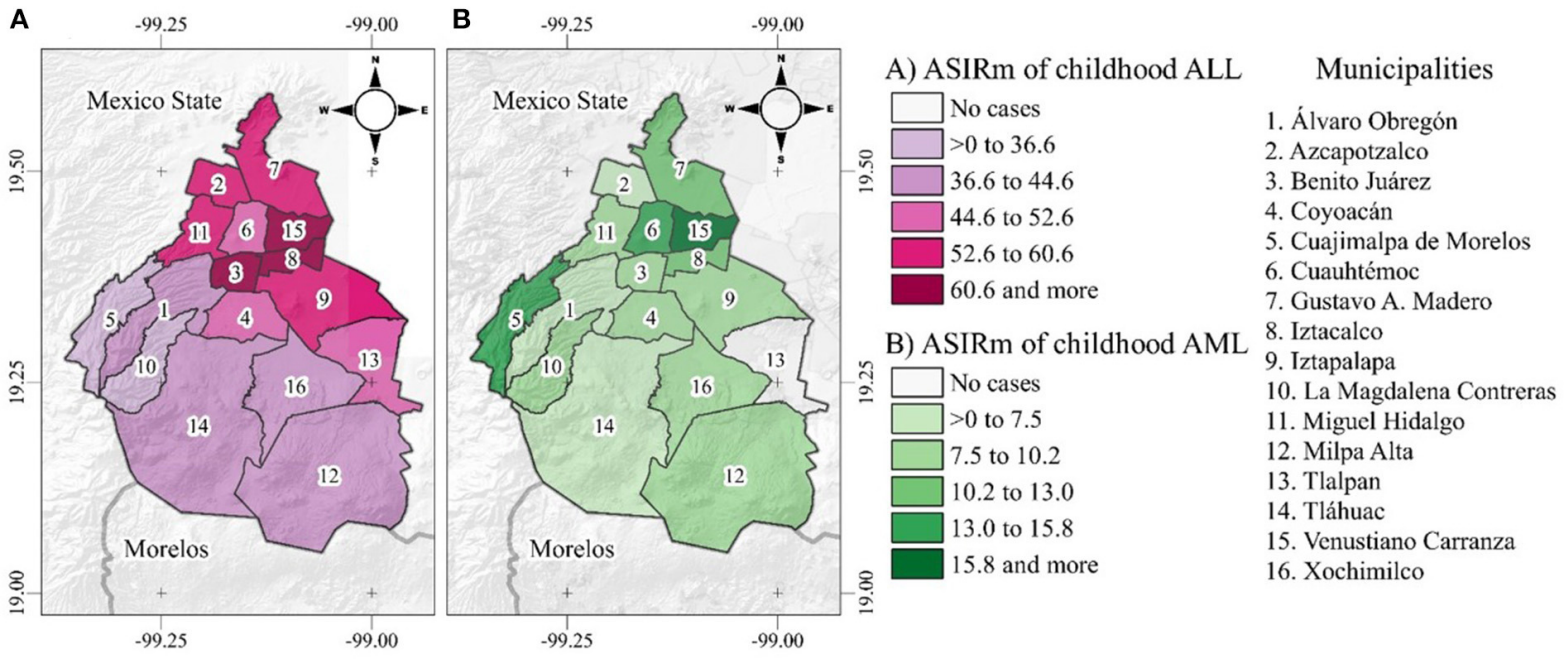
**(A)** MC ASIRm Maps. The municipalities are alphabetically ordered. ALL ASIRm, age-standardized incidence rates by municipalities. **(B)** MC ASIRm Maps. The municipalities are alphabetically ordered. AML ASIRm, age-standardized incidence rates by municipalities.

Figure 2 shows the ASIRm in cases per million. The highest ASIRm values for ALL were found in the municipalities of Iztacalco (68.6), Venustiano Carranza (66.6), and Benito Juárez (62.8) (Figure 2A). The highest ASIRm for AML was found in Venustiano Carranza (18.6) (Figure 2B). Notably, the highest ASIRm values for the ALL and AML subtypes were found in the Central and Eastern zones of MC.

### AL trends

The AAPC results did not show statistical evidence of an increasing or decreasing trend (*p* <0.05). Notably, only the ALAL subgroup was analyzed using the technique of rate imputation; analysis of the other subgroups was not possible due to the small number of patients. On the other hand, given the fact that we did not find joinpoints throughout the data series, the most appropriate way to refer to the trend summary measure is APC (annual percent change). Then the results displayed in Table 1 refer to APC.

### AL incidence rate by age

The highest cAIR for ALL was observed in the 1–4 year age group (80.5 cases per million), where the cAIR for ALL was higher among males (88.1) than for females (72.6). In contrast, within the 5–9 year age group, the cAIR for ALL was higher among females than males. The highest cAIR values for AML were observed in the 1–4 year age group (11.5) and in the 10–14 year age group (10.4). The cAIR for AML was higher among males than females (Table 1).

### AL incidence rate by immunophenotype

The ASIRw was 47.3 (cases per million) for the B-cell subtype, and 3.7 for the T-cell subtype (Table 1).

### Peak age of incidence of AL subtypes

**Figure 3:**
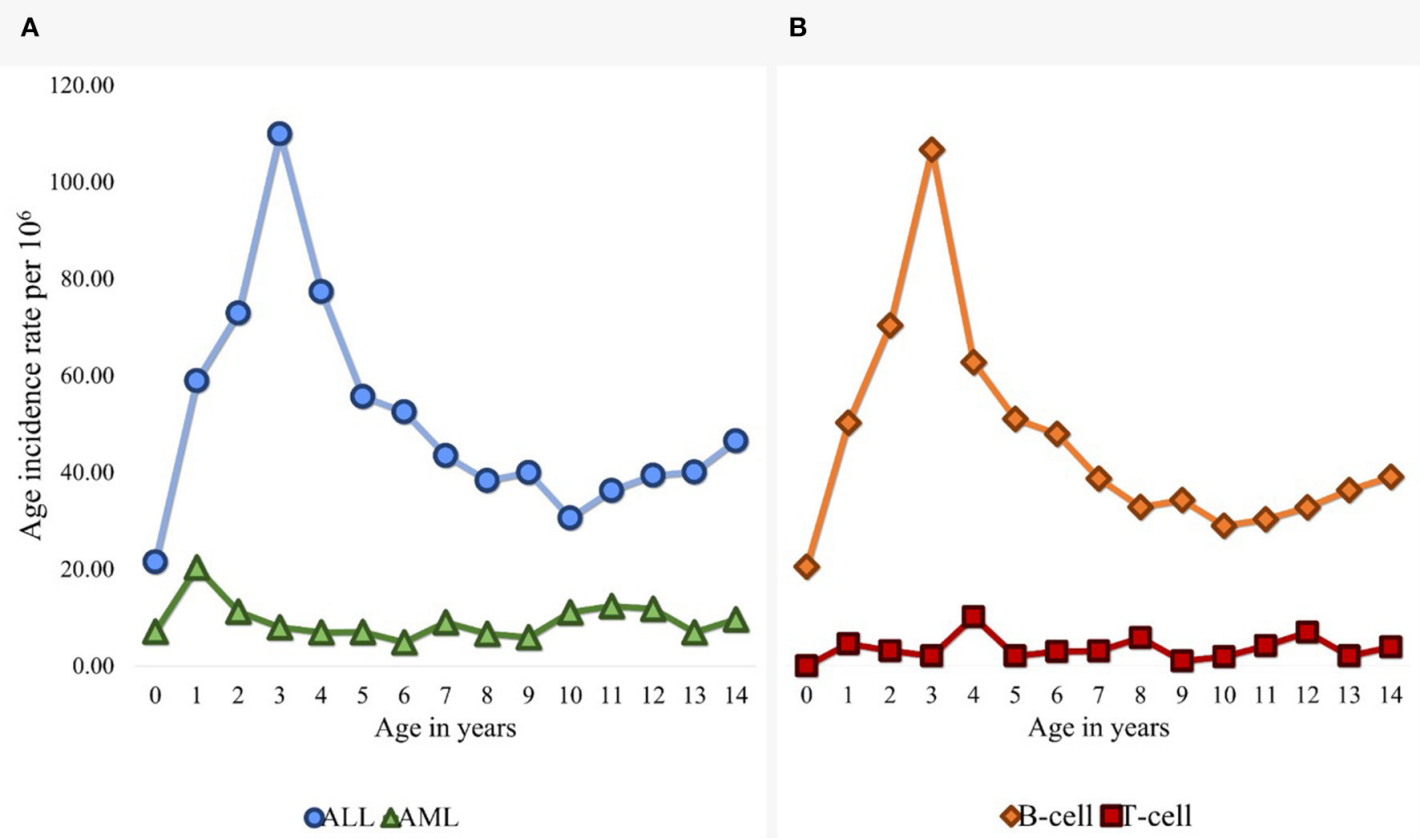
**(A)** Age-standardized incidence rates of childhood AL in MC (2010–2017) by subtype: ALL and AML. **(B)** Age-standardized incidence rates of childhood AL in MC (2010–2017) by immunophenotype: B-cell and T-cell ALL.

Among cases of ALL, we observed the peak incidence at 3 years old, with a decrease at 10 years of age, and then a slight increase at 14 years old. Among cases of AML, we observed a peak incidence at 1 year old, with a slight increase between 10 and 12 years old (Figure 3A). No age-related pattern was observed for the OL subtype (not showed). For B-cell, we observed a major incidence age peak at 3 years old, and a slight increase at 14 years old. For T-cell AL, we observed multiple incidence age peaks at one, four, eight, and 12 years old (Figure 3B).

## Discussion

In this study, we determined the AL incidence rates in the population under 15 years of age from MC during the period of 2010–2017. The ASIRw of AL was slightly higher (63.3 cases per million) than those previously reported in studies conducted in MC: 58.4 during 1996–2000; 55.4 during 1996–2002; 62.2 during 1996–2013 and 57.6 during the period 2006–2007 (3, 8–10). This finding is consistent with the observed among Hispanic children living in the USA (65.4), Texas (59.8) and California (55.0) (2, 7, 78). Notably, the North American regions with Mexican, Canadian, and American populations display high incidence rates of AL (31.4–65.4) in comparison to other populations (2, 3, 8–10, 72, 78–81). Furthermore, studies in children of Hispanic ancestry, aged 0–19 years, have also shown incidence rates that are among the highest worldwide (7, 11, 82–89) (Supplementary Table 1).

Several studies show high AL incidence rates among Oceanians (Pacific Islanders) (56.4) (2), Australians (50.0–53.5) (90–92), and Europeans (42.1–57.1) (93–101); but these rates are slightly below than those reported for populations in the North American regions with Hispanic ancestry.

The incidence rates in Asia (30.7–53.5) (102–109) and African countries (0.5–33.6) (2, 110, 111) are appreciably lower than the rates reported in our study and worldwide. However, the rates reported from Africa should be interpreted cautiously, because it has been mentioned that the cancer registries of some African countries are likely affected by under-reporting (112).

Interestingly, in South America, Central America, and the Caribbean regions, which are home to children of Latin American ancestry, show lower AL rates (33.3–49.8) than those reported in the present research (2, 113, 114). One possible explanation is the fact that in the Caribbean subregion, the African ancestry is also prevalent, which has been associated with a low incidence rate of childhood AL. On the other hand, in the southernmost regions of South America and Costa Rica in Central America, the predominant component of the ancestry is European, which has been associated with moderate incidence rates of childhood AL instead of high rates (115). However, other relevant factors should be taken into consideration for explaining these differences, such as the quality of the cancer registries, the environmental, lifestyle factors among others.

### AL main subtypes

#### ALL

Previous studies conducted in MC had already reported high incidence rates for childhood ALL during the periods 1996–2000 (44.9), 1996–2002 (43.2), and 2006–2007 (49.5), respectively. However, for the present study, a higher incidence rate than previously reported (53.1) was observed. It should be noted that all of these rates are among the highest in the world; nevertheless, to our knowledge, the incidence observed in the present study is one of the first reported above 50 cases per million in MC and globally. Additionally, the rate of incidence of ALL in MC is consistent with the reported in the population with Hispanic ancestry (116–118). This increase may not only be explained by genetic susceptibility but also with the interaction with the exposure to some environmental factors (119).

#### AML

The ASIRw for AML was 9.4 cases per million in the present investigation. The incidence rate for this subtype of AL has remained stable and similar to that reported in previous studies conducted in MC throughout the periods of 1996–2000 (10.6), 1996–2002 (9.8), 2006–2007 (6.9) and 2010–2014 (7. 7) (3.8–10.6); as well as have been consistent to the rates reported worldwide in the regions of North America (7.0–10.6) (3, 8, 9, 72, 78, 79, 120); Europe (5.9–9.3) (94, 95, 99–101); Australia (7.2–8.7) (90, 91) and South America, Central America, and Caribbean (8.3–9.2) (112, 113); with the exception of the regions of Asia (5.4) (104) and Africa (0.3 to 6.0) (111). It has also been observed that AML occurs more frequently in infants and adolescents (121), in contrast to the ALL subtype. Moreover, several investigations support that the AML subtype present different etiopathogenic components compared to the subgroup of patients with ALL (122). However, the etiology for both leukemias remains unresolved until date, but, a small number of risk factors have been reported as established for the development of AML in children (121, 123–131).

### AL incidence trends

During the period analyzed in the present work (2010–2017), no trends for AL incidence rates were observed. This is similar to the reported in MC from 2002 to 2013 where a phenomenon of stability in incidence rates for childhood AL had been described. Nonetheless, it should be noted that previous studies included data from a single health institution (IMSS) in contrast with the present research which was conducted in all public health institutions that care for children with cancer (IMSS, ISSSTE, SS, SSCDMX). This could support that the results obtained in the present study are representative for this disease in MC. On the other hand, this stable pattern also suggests that incidence rates for AL have remained high for at least over the last two decades in the population of MC (10). Moreover, the stability of the incidence rates described in this report is not consistent with those published in children of Hispanic ancestry in the USA, where the increasing trend is higher in comparison to other ethnic groups (Amerindian, Asian-Pacific, White, Afro-American) (12). A longer evaluation may be required.

The frequency of B-cell (88.7%) in our study is higher than the reported previously in MC (73.2–85.1%) (3, 15, 24, 132) but is similar to worldwide reported frequencies (80–92%) (111, 112), except to that reported in Asian countries (92.7%) (132). These differences could be associated with the genetic profile of our population with B-cell, as reported in a study by Moreno-Lorenzana et al. (133), who report a high frequency (54.1%) of dominant-negative *IKZF1* isoform transcripts in a pediatric population from a single medical institution (133). One of the reasons why there is a greater frequency of B-cell among Hispanics may be its relationship with *IKZF1* and *ARID5B*, which have also been reported more frequently in Hispanics. In this regard, it has been described that both the Hispanic ancestry and the presence of the germline *IKZF1* variant are related to a poor response to treatment in children with B-cell (134). As well as it has been reported the role of *ARID5B* gene in B-cell development, particularly, in the Hispanic population (135, 136). Recently, the *ARID5B* gene has been associated with an increased risk of B-cell in Mexican children (137, 138).

In the present study, the frequency of T-cell ALL was of 7.0%. This finding is similar to the reported in the most of the studies conducted in MC (5.7–12.4%) (3, 24, 25, 139) and other regions of the world (10–15%) (140, 141). Conversely, in another study conducted in MC by Perez-Saldivar et al., where the authors reported a frequency around the 24% among ALL subtypes (3). Noteworthy, an elevated frequency of T-cell leukemias has also been reported in other developing countries (18–23.4%) (142, 143). It is well-recognized that the T-cell subtype is associated with a poor prognosis of the disease, particularly, a high incidence of isolated central nervous system relapse has been described in this group (144).

Regarding the age-specific incidence of ALL, previous studies conducted in MC have reported the existence of a maximum age peak in ALL occurring at the age of 3 years, which is consistent with the observed in the present investigation and which falls within the peak age range reported for ALL in international studies (2–5 years) (145). We also found a slight increase in incidence at 14 years of age, which was also observed in MC by Bernaldez-Rios et al. (15). Until this moment, we do not have an explanation for this last increase during adolescence but it has been observed that in countries where there is a lower frequency of the *ETV6-RUNX1* rearrangement there is a higher frequency of ALL in adolescents and also a higher frequency of gene rearrangements with worse prognosis (146). In addition, some genetic factors such as the *Ph-like* phenotype may be one of the reasons for this observation due to the fact that its prevalence increase with the age in ALL patients. Nonetheless, this hypothesis should be studied further in Mexican population.

### Frequency of ALL by gene rearrangements

The overall detection of the four most common gene rearrangement was low as it has been reported in previous studies conducted in MC which also have reported a low frequency of *ETV6-RUNX1*, ranging from 7.4 to 13.5% (26, 27, 64), which is consistent with the findings of the present study (8.2%) and other Hispanic populations (27, 147) but lower than the frequency reported in developed countries (25–30%) (25, 29). Moreover, the low prevalence of *ETV6-RUNX1* has implications in the ancestry component of the childhood ALL. In this regard, it has been described that when the Amerindian (indigenous) ancestry is higher, the *ETV6-RUNX1* is less common. This type of ancestry has been consistently associated with a high incidence and poor prognosis of childhood ALL and in a recent study was reported that the Amerindian is the most prevalent ancestry in pediatric population of MC (148, 149). Until date, the reasons behind this relationship has not been established and further investigation is required for explaining the high incidence and mortality rates observed in Hispanic populations (150).

On the other hand, pediatric cases with ALL attended in public hospitals of MC consistently have displayed a higher frequency of the *TCF3-PBX1* rearrangement (7.1–11.5%), in comparison to the reported in the worldwide literature in children with B-cell (5%) (26, 27). In the present study, this frequency was appreciably lower (2.5%) than previously reported. Our finding could be due to the fact that at the beginning of the study not all participating hospitals sent the leukemia patients' samples for analysis. This assumption is supported by a recent study of our research group where this gene rearrangement was found in 7.5% of ALL pediatric cases such as it has been previously reported (42). In addition, the prevalence of the other gene rearrangements that were analyzed in the present research were similar to the reported in MC and worldwide (1–3%) (42).

### AL incidence rates by municipalities

On the other hand, the highest ASIRm rates for the AL subtypes were predominantly concentrated among the municipalities of Venustiano Carranza, Iztacalco, Iztapalapa, and Benito Juárez. In particular, Venustiano Carranza showed higher rates of all AL subtypes compared to the other municipalities. Notably, the MC International Airport (AICM) is located in this municipality, which could increase children's exposure to air pollution. It has been suggested that exposure to pollution generated at airports is a possible environmental risk factor for AL development (151). However, further studies are required to determine if this factor contributes to the AL incidence considering that other population with high levels of air pollution did not show the highest incidence rates worldwide. Importantly, MC generally has highly harmful levels of air pollution (152–155) due to its geographic, climatic, and social conditions (49, 156). Prior studies have reported that living in an urban area or near an industrial zone (157–159) and being exposed to either vehicular traffic (160) or benzene derivatives (161, 162), are factors potentially contributing to the development of childhood AL (126, 163–166). On the other hand, we observed a relatively lower AL incidence rates in the municipalities located south and east of MC, such as Milpa Alta, Xochimilco, and Tláhuac, which are characterized by preserved areas of agricultural land production (60–62). Overall, the presently observed AL incidence rates are among the highest that have been reported in our country, and our findings highlight the predominance of this disease in the most urbanized municipalities. Notably, there remains controversy regarding international studies that have evaluated and compared the level of risk between urban and rural areas (159, 162, 167).

Another factor that must be considered is the role of ethnicity. In American countries, the highest AL incidence rates have been reported in children with an Amerindian (indigenous) ancestry (168). Quiroz et al. emphasized that mestizo countries, such as Mexico, Ecuador, and Colombia, had the highest pediatric AL incidence rates (40–57 cases per million) among the countries included. Conversely, to those American countries where the population was mainly from an European ancestry (e.g., the United States, Argentina, and Chile) exhibiting lower incidence rates (33–37) such as the reported for European populations (168). Notably, in Brazil, three zones have been identified: the zone with a greater indigenous presence exhibits the highest incidence rate (over 40), the zone with a larger European population presenting an intermediate incidence rate (~30), and the zone with a larger African ancestry population which have exhibited the lowest incidence for AL (20) (168, 169). The same phenomenon has been observed among White, Hispanic, and Afro-American populations from the United States (2, 7, 11, 79–81). This has also been reported in Mexico. For instance, in south Mexico, where the population is mostly of Amerindian (indigenous) ancestry, the incidence rates have been reported over the 70 cases per million, while in the north, where the population mainly has an European ancestry, the incidence rates (32) are similar to those reported in the White population in the United States (9, 170).

Notably, the geographical distribution of the ethnic groups and the socioeconomic status (SES) within populations is also relevant. In the present study the highest rates of AL were observed in municipalities located mainly at MC east (Venustiano Carranza, Iztacalco, and Iztapalapa), which are characterized by a low SES. Conversely, low incidence rates of AL were also observed in municipalities with a better SES (La Magdalena Contreras and Cuajimalpa de Morelos). SES cannot be separated from the ethnic composition, the lower the SES of a Mexican individual, the higher the probability to have an Amerindian ancestry (171). For instance, it has been reported in Mexico that the individuals with the darker skin colors also show significantly lower levels of education, lower remunerated jobs and higher probabilities of living in poverty conditions in comparison to people with lighter hues (172, 173).

### Limitations and strengths of the study

One limitation of the present research was not to have included the children diagnosed and attended in private hospitals. Nonetheless, the main strength of the study is including nearly all cases of childhood leukemia and being able to include genetic data. Cases were recruited from public hospitals of MC in which an estimated 97.5% of children with leukemia who reside in MC are attended (64). Furthermore, the standardized incidence rates were obtained similarly to the previous report by our group during 2006–2007 in MC (3) considering characteristics such as the population, geography, health institutions and age groups. With respect to the previous study, in the present investigation we observed higher rates of ALL and AML. However, the APC results showed no trend for any AL subtype.

To our knowledge, this is the first article to actively study the incidence of childhood AL by using information from all public health institutions in MC during the research period of 2010–2017.

## Conclusion

The incidence rate of childhood AL in MC continued to be among the highest in the world, at over 60 cases per million. Additionally, the results highlight the heterogeneous distributions of the incidence rates among different municipalities of MC. These data suggest possible roles of environmental, epigenetic, or lifestyle factors in AL development among Mexican children. It is important to conduct further research studies to examine the relationship between these factors and the risk of AL in these areas.

## Data availability statement

The raw data supporting the conclusions of this article will be made available by the authors, without undue reservation.

## Author contributions

JF-L, DD-R, JM-A, and JN-E: conceptualization. JF-L, DD-R, and JM-A: methodology. JF-L, DD-R, JM-A, and AA-L: formal analysis. JF-L and DD-R: investigation and writing—original draft preparation. EJ-H, JM-T, JP-G, AA-L, MP-S, AM-S, JT-N, KS-L, LF-V, RE-E, RA-S, MV-A, LM-P, NN-V, AG-Á, MC-M, MA-I, VB-M, RC-C, SJ-M, RR-L, HR-V, NL-S, AR-L, AH-M, EV, MM-R, OS-R, and JA-G: resources. JF-L, DD-R, JN-E, and JM-A: writing—review and editing, supervision, and funding acquisition. All authors reviewed the final manuscript, read, and approved the submitted version.

## Funding

This work was supported by the Consejo Nacional de Ciencia y Tecnología [Grant Numbers: FORDECYT-PRONACES: 303019; CB 2015-1-258042-M; R-2020-785-022, FONCICYT/37/2018 and FIS/IMSS/PRIO/15/048], and Secretaría de Educación, Ciencia, Tecnología e Innovación (México) SECTEI/203/2019.

## Conflict of interest

The authors declare that the research was conducted in the absence of any commercial or financial relationships that could be construed as a potential conflict of interest.

## Publisher's note

All claims expressed in this article are solely those of the authors and do not necessarily represent those of their affiliated organizations, or those of the publisher, the editors and the reviewers. Any product that may be evaluated in this article, or claim that may be made by its manufacturer, is not guaranteed or endorsed by the publisher.
