# Supplementary material for: Persistently high incidence rates of childhood acute leukemias from 2010 to 2017 in Mexico City: A population study from the MIGICCL
Source: Front Public Health. 2022 Sep 14;10:918921. doi: 10.3389/fpubh.2022.918921 (PMC9518605; doi:10.3389/fpubh.2022.918921)
Supplement: Supplementary file 1 [file Table_1.pdf]

SUPPLEMENTARY TABLE 1. Comparison of Age-Standardized Incidence Rates of All Childhood Acute Leukemias Per Million

World Region	Country	Province/Region	Ethnic Group	Age range years	Period study	ASIR AL	ASIR ALL	ASIR AML	Authors
North America	USA	Nationwide	White Hispanic American	0–14	2001–2010	65.4	-	-	(Steliarova-Foucher et al. 2017)
**North America**	**Mexico**	**Mexico City (present study)**	**Mexican**	**0–14**	**2010–2017**	**63.3**	**53.1**	**9.4**	
North America	Mexico	Mexico City	Mexican	0–14	1996–2013	62.2	-	-	(Fajardo-Gutiérrez et al. 2016)
North America	USA	Florida	Hispanics American	0–14	1985–1997	-	49.7	-	(Wilkinson et al. 2001)
North America	USA	Texas	American	0–14	2008–2017	59.8	44.4	7.8	(Services 2020)
North America	Mexico	Mexico City	Mexican	0–14	1996–2000	58.4	44.9	10.6	(Mejía-Aranguré et al. 2005)
North America	Mexico	Mexico City	Mexican	0–14	2006–2007	57.6	49.6	6.9	(Pérez-Saldivar et al. 2011)

ASIR, age-standardized incidence rates; AL, acute leukemias; ALL, acute lymphoblastic leukemia; AML, acute myeloblastic leukemia. *Crude Incidence Rates

Cont. SUPPLEMENTARY TABLE 1. Comparison of Age-Standardized Incidence Rates of All Childhood Acute Leukemias Per Million

World Region	Country	Province/Region	Ethnic Group	Age range years	Period study	ASIR AL	ASIR ALL	ASIR AML	Authors
North America	Mexico	Mexico City	Mexican	0–14	1996–2002	57.6	43.2	9.8	(Fajardo-Gutiérrez et al. 2007)
North America	Mexico	Mexico City	Mexican	0–14	2010–2014	-	-	7.7	(Mejía-Aranguré et al. 2016)
North America	USA	SEER	American	0–14	2005–2014	-	-	7.6	(X. Chen et al. 2019)
North America	USA	Nationwide	Oceania American	0–14	2001–2010	56.4	-	-	(Steliarova-Foucher et al. 2017)
North America	Canada	Canada	Canadian	0–14	2001–2010	55.5	-	-	(Steliarova-Foucher et al. 2017)
North America	USA	Nationwide	White non-Hispanic American	0–14	2001–2010	54.5	-	-	(Steliarova-Foucher et al. 2017)
North America	USA	California	Hispanics American	0–14	1988–1994	54.0	44.0	7.0	(Glazer et al. 1999)

ASIR, age-standardized incidence rates; AL, acute leukemias; ALL, acute lymphoblastic leukemia; AML, acute myeloblastic leukemia. *Crude Incidence Rates

Cont. SUPPLEMENTARY TABLE 1. Comparison of Age-Standardized Incidence Rates of All Childhood Acute Leukemias Per Million

World Region	Country	Province/Region	Ethnic Group	Age range years	Period study	ASIR AL	ASIR ALL	ASIR AML	Authors
North America	USA	Nationwide	Southeast Asian American	0–14	2001–2010	52.7	-	-	(Steliarova-Foucher et al. 2017)
North America	USA	Nationwide	Southern European American	0–14	2001–2010	51.1	-	-	(Steliarova-Foucher et al. 2017)
North America	USA	Nationwide	Western European Americans	0–14	2001–2010	50.7	-	-	(Steliarova-Foucher et al. 2017)
North America	USA	Nationwide	Northern European American	0–14	2001–2010	49.1	-	-	(Steliarova-Foucher et al. 2017)
North America	USA	Nationwide	East Asian American	0–14	2001–2010	47.4	-	-	(Steliarova-Foucher et al. 2017)
North America	USA	Florida	Non-Hispanic White American	0–14	1985–1997	-	38.7	-	(Wilkinson et al. 2001)
North America	USA	Nationwide	Non-Hispanic White American	0–14	1988–1994	46.0	38.0	7.0	(Glazer et al. 1999)

ASIR, age-standardized incidence rates; AL, acute leukemias; ALL, acute lymphoblastic leukemia; AML, acute myeloblastic leukemia. *Crude Incidence Rates

Cont. SUPPLEMENTARY TABLE 1. Comparison of Age-Standardized Incidence Rates of All Childhood Acute Leukemias Per Million

World Region	Country	Province/Region	Ethnic Group	Age range years	Period study	ASIR AL	ASIR ALL	ASIR AML	Authors
North America	USA	Nationwide	West Asian American	0–14	2001–2010	45.2	-	-	(Steliarova-Foucher et al. 2017)
North America	USA	Nationwide	Eastern European American	0–14	2001–2010	44.3	-	-	(Steliarova-Foucher et al. 2017)
North America	USA	Nationwide	Asian and Pacific Islanders American	0–14	2001–2010	43.9	-	-	(Steliarova-Foucher et al. 2017)
North America	USA	Nationwide	South Asian and Indian Americans	0–14	2001–2010	34.1	-	-	(Steliarova-Foucher et al. 2017)
North America	USA	Nationwide	Native American	0–14	2001–2010	31.5	-	-	(Steliarova-Foucher et al. 2017)
North America	USA	Nationwide	African-American	0–14	2001–2010	31.3	-	-	(Steliarova-Foucher et al. 2017)
North America	USA	Florida	African American	0–14	1985–1997	-	21.3	-	(Wilkinson et al. 2001)

ASIR, age-standardized incidence rates; AL, acute leukemias; ALL, acute lymphoblastic leukemia; AML, acute myeloblastic leukemia. *Crude Incidence Rates

Cont. SUPPLEMENTARY TABLE 1. Comparison of Age-Standardized Incidence Rates of All Childhood Acute Leukemias Per Million

World Region	Country	Province/Region	Ethnic Group	Age range years	Period study	ASIR AL	ASIR ALL	ASIR AML	Authors
Europe	Switzerland	Nationwide	Swiss	0–14	2005–2014	57.1	-	-	(Sommer et al. 2019)
Europe	Italia	Nationwide	Italian	0–14	2003–2008	56.1 male, 51.6 female	43.5	6.8	(AIRTUM Working Group, CCM, and AIEOP Working Group 2016)
Europe	Hungary	Nationwide	Hungarian	0–14	1971–2015	56.0	39.2	9.3	(Jakab et al. 2017)
Europe	Czech Republic	Nationwide	Czech	0–14	1994–2016	52.3	-	-	(Krejci et al. 2020)
Europe	Spain	Nationwide	Spaniard	0–14	1983–2002	45.9	-	-	(Peris-Bonet et al. 2010)
Europe	Italy	Sardinia	Sardinian	0–14	1974–2003	---	38.5	-	(Broccia et al. 2020)
Europe	Netherlands	Nationwide	Dutch	0–14	1990–2017	44.8	35.0	7.8	(Reedijk et al. 2020)

ASIR, age-standardized incidence rates; AL, acute leukemias; ALL, acute lymphoblastic leukemia; AML, acute myeloblastic leukemia. *Crude Incidence Rates

Cont. SUPPLEMENTARY TABLE 1. Comparison of Age-Standardized Incidence Rates of All Childhood Acute Leukemias Per Million

World Region	Country	Province/Region	Ethnic Group	Age range years	Period study	ASIR AL	ASIR ALL	ASIR AML	Authors
Europe	Spain	Castilla and León	Spaniard	0–14	2003–2014	44.5	37.7	5.9	(González-García et al. 2018)
Europe	Estonia	Nationwide	Estonian	0–14	1995–2016	42.1	31.4	7.4	(Paapsi et al. 2020)
Australia	Australia	Nationwide	Australian	0–14	1983–2015	53.5	42.5	7.2	(Youlden et al. 2020)
Australia	Australia	Nationwide	Australian	0–14	1983–2006	53.1	40.8	8.7	(Baade et al. 2010)
Australia	Australia	Queensland	Australian	0–14	2007–2016	50.0	-	-	(Holland et al. 2020)
Asia	Iraq	Basrah	Iraqi	0–14	2012–2016	53.5 male, 43.6 female	-	-	(Al-Asadi and Ibrahim 2018)
Asia	Iran	Nationwide	Iranian	0–14	1990–2016	48.2	-	-	(Shabani et al. 2020)

ASIR, age-standardized incidence rates; AL, acute leukemias; ALL, acute lymphoblastic leukemia; AML, acute myeloblastic leukemia. *Crude Incidence Rates

Cont. SUPPLEMENTARY TABLE 1. Comparison of Age-Standardized Incidence Rates of All Childhood Acute Leukemias Per Million

World Region	Country	Province/Region	Ethnic Group	Age range years	Period study	ASIR AL	ASIR ALL	ASIR AML	Authors
Asia	Iran	Basra	Iranian	0–14	2017	47.0	-	-	(Abood, Abdahmed, and Mazyed 2020)
Asia	Saudi Arabia	Nationwide	Saudi	0–14	2005–2009	35.3	25.8	5.4	(Belgaumi et al. 2019)
Asia	Thailand	Khon Kaen	Thai	0–14	1985–2009	33.8	-	-	(Wiangnon et al. 2014)
Asia	Kuwait	Nationwide	Kuwaiti	0–14	1980–2014	30.7	-	-	(Akhtar, Al-Abkal, and Al-Shammari 2020)
Asia	Iran	Nationwide	Iranian	0–14	2006–2014	---	22.5	-	(Rahimi Pordanjani et al. 2020)
Asia	Saudi Arabian	Nationwide	Saudi	0–14	2001–2014	---	23.5	-	(Jastaniah et al. 2020)
South America, Central America, and the Caribbean	South America, Central America, and the Caribbean	South America, Central America, and the Caribbean	South American	0–14	2001-2010	49.8	-	-	(Steliarova-Foucher et al. 2017)

ASIR, age-standardized incidence rates; AL, acute leukemias; ALL, acute lymphoblastic leukemia; AML, acute myeloblastic leukemia. *Crude Incidence Rates

Cont. SUPPLEMENTARY TABLE 1. Comparison of Age-Standardized Incidence Rates of All Childhood Acute Leukemias Per Million

World Region	Country	Province/Region	Ethnic Group	Age range years	Period study	ASIR AL	ASIR ALL	ASIR AML	Authors
South America, Central America, and the Caribbean	Argentina	Nationwide	Argentinian	0–14	2000–2008	47.5	37.7	9.2	(Moreno et al. 2013)
South America, Central America, and the Caribbean	South America, Central America, and the Caribbean	South America, Central America, and the Caribbean	Central American and Caribbean	0–14	2001–2010	45.5	-	-	(Steliarova-Foucher et al. 2017)
South America, Central America, and the Caribbean	Brazil	Campinas	Brazilian	0–14	1996–2005	33.3	24.2	8.3	(Oliveira Friestino et al. 2018)
Africa	Mauritius	Nationwide	Mauritianian	0–14	2003–2012	33.6*	11.1*	3.8*	(Stefan et al. 2017)
Africa	France	Reunion	Réunioneses	0–14	2002–2008	32.0*	24.7*	6*	(Stefan et al. 2017)
Africa	South African Republic	Private health sector	South African	0–14	2008–2017	30.2	-	-	(Otoo et al. 2020)
Africa	Ethiopia	Addis Ababa	Ethiopian	0–14	2011–2013	28.8*	22.3*	2.3*	(Stefan et al. 2017)

ASIR, age-standardized incidence rates; AL, acute leukemias; ALL, acute lymphoblastic leukemia; AML, acute myeloblastic leukemia. *Crude Incidence Rates

Cont. SUPPLEMENTARY TABLE 1. Comparison of Age-Standardized Incidence Rates of All Childhood Acute Leukemias Per Million

World Region	Country	Province/Region	Ethnic Group	Age range years	Period study	ASIR AL	ASIR ALL	ASIR AML	Authors
Africa	Africa	North Africa	North African	0–14	2001–2010	28.1	-	-	(Steliarova-Foucher et al. 2017)
Africa	South African Republic	Nationwide	White South African	0–14	2008–2012	27.3*	22.9*	3.1*	(Stefan et al. 2017)
Africa	Kenya	Eldoret	Kenyan	0–14	2007–2011	24.3*	15.1*	4.3*	(Stefan et al. 2017)
Africa	Kenya	Nairobi	Kenyan	0–14	2007-2012	21.0*	14.7*	2.5*	(Stefan et al. 2017)
Africa	Zimbabwe	Harare	Zimbabwean	0–14	2003–2012	13.2*	6.6*	2.1*	(Stefan et al. 2017)
Africa	Africa	Sub-Saharan Africa	Sub-Saharan African	0–14	2001–2010	12.1	-	-	(Steliarova-Foucher et al. 2017)
Africa	Uganda	Kyadondo County	Ugandan	0–14	2003–2012	12.2*	5.8*	1.4*	(Stefan et al. 2017)

ASIR, age-standardized incidence rates; AL, acute leukemias; ALL, acute lymphoblastic leukemia; AML, acute myeloblastic leukemia. *Crude Incidence Rates

Cont. SUPPLEMENTARY TABLE 1. Comparison of Age-Standardized Incidence Rates of All Childhood Acute Leukemias Per Million

World Region	Country	Province/Region	Ethnic Group	Age range years	Period study	ASIR AL	ASIR ALL	ASIR AML	Authors
Africa	South African Republic	South African Republic	South African	0–14	2008–2012	11.7*	8.0*	3.2*	(Stefan et al. 2017)
Africa	Botswana	Botswana	Botswanese	0–14	2003–2008	11.6*	6.6*	3.9*	(Stefan et al. 2017)
Africa	South African Republic	Nationwide	Black South African	0–14	2008–2012	9.2*	5.7*	3.1*	(Stefan et al. 2017)
Africa	Mali	Bamako	Malian	0–14	2006–2014	8.5*	5.8*	1.0*	(Stefan et al. 2017)
Africa	Malawi	Blantyre	Malawian	0–14	2003–2010	6.3*	0.3*	0.9*	(Stefan et al. 2017)
Africa	Niger	Niamey	Nigerian	0–14	2001–2009	4.5*	3.0*	-	(Stefan et al. 2017)
Africa	Nigeria	Ibadan	Nigerian	0–14	2003–2012	3.5*	1.2*	1.2*	(Stefan et al. 2017)

ASIR, age-standardized incidence rates; AL, acute leukemias; ALL, acute lymphoblastic leukemia; AML, acute myeloblastic leukemia. *Crude Incidence Rates

Cont. SUPPLEMENTARY TABLE 1. Comparison of Age-Standardized Incidence Rates of All Childhood Acute Leukemias Per Million

World Region	Country	Province/Region	Ethnic Group	Age range years	Period study	ASIR AL	ASIR ALL	ASIR AML	Authors
Africa	South African Republic	Eastern Cape	Eastern Cape	0–14	2003–2012	3.2*	0.5*	-	(Stefan et al. 2017)
Africa	The Gambia	Nationwide	Gambian	0–14	2002–2011	2.6*	0.9*	-	(Stefan et al. 2017)
Africa	Guinea	Conakry	Guinean	0–14	2001–2010	0.5*	-	0.3*	(Stefan et al. 2017)
International population	International population	International population	International population	0–14	2001–2010	46.4	-	-	(Steliarova-Foucher et al. 2017)
North America	USA	Nationwide	Oceanian (Guamanian, Samoan, Tongan) American	0–19	1998–2002 and 2003–2007	64.8	33.9	30.9	(Moore et al. 2020)
North America	USA	Nationwide	Hispanic-White American	0–19	1992–2013	60.5	48.9	9.7	(Barrington-Trimis et al. 2017)
North America	USA	California	Hispanic American	0–19	1990–2012	55.0	44.5	8.2	(Giddings et al. 2016)

ASIR, age-standardized incidence rates; AL, acute leukemias; ALL, acute lymphoblastic leukemia; AML, acute myeloblastic leukemia. *Crude Incidence Rates

Cont. SUPPLEMENTARY TABLE 1. Comparison of Age-Standardized Incidence Rates of All Childhood Acute Leukemias Per Million

World Region	Country	Province/Region	Ethnic Group	Age range years	Period study	ASIR AL	ASIR ALL	ASIR AML	Authors
Africa	South African Republic	Eastern Cape	Eastern Cape	0–14	2003–2012	3.2*	0.5*	-	(Stefan et al. 2017)
Africa	The Gambia	Nationwide	Gambian	0–14	2002–2011	2.6*	0.9*	-	(Stefan et al. 2017)
Africa	Guinea	Conakry	Guinean	0–14	2001–2010	0.5*	-	0.3*	(Stefan et al. 2017)
International population	International population	International population	International population	0–14	2001–2010	46.4	-	-	(Steliarova-Foucher et al. 2017)
North America	USA	Nationwide	Oceanian (Guamanian, Samoan, Tongan) American	0–19	1998–2002 and 2003–2007	64.8	33.9	30.9	(Moore et al. 2020)
North America	USA	Nationwide	Hispanic-White American	0–19	1992–2013	60.5	48.9	9.7	(Barrington-Trimis et al. 2017)
North America	USA	California	Hispanic American	0–19	1990–2012	55.0	44.5	8.2	(Giddings et al. 2016)

ASIR, age-standardized incidence rates; AL, acute leukemias; ALL, acute lymphoblastic leukemia; AML, acute myeloblastic leukemia. *Crude Incidence Rates

Cont. SUPPLEMENTARY TABLE 1. Comparison of Age-Standardized Incidence Rates of All Childhood Acute Leukemias Per Million

World Region	Country	Province/Region	Ethnic Group	Age range years	Period study	ASIR AL	ASIR ALL	ASIR AML	Authors
North America	USA	CDC	Hispanic American	0–19	2001–2008	-	42.9	-	(Siegel et al. 2017)
North America	USA	Nationwide	Asian Indian/Pakistani American	0–19	1998–2002	50.1	33.2	8.5	(Moore et al. 2020)
North America	USA	CDC	American	0–19	2003–2014	47.5	-	-	(Siegel et al. 2017)
North America	USA	Nationwide	East Asian (Chinese, Japanese Korean) American	0–19	1998–2002	47.2	34.8	8.2	(Moore et al. 2020)
North America	USA	Nationwide	Non-Hispanic White American	0–19	1992–2013	44.5	34.4	8.7	(Barrington-Trimis et al. 2017)
North America	USA	CDC	White American	0–19	2001–2014	-	34.2	-	(Siegel et al. 2017)
North America	USA	Nationwide	Non-Hispanic White American	0–19	1988–2002	44.3	34.0	8.2	(Moore et al. 2020)

ASIR, age-standardized incidence rates; AL, acute leukemias; ALL, acute lymphoblastic leukemia; AML, acute myeloblastic leukemia. *Crude Incidence Rates

Cont. SUPPLEMENTARY TABLE 1. Comparison of Age-Standardized Incidence Rates of All Childhood Acute Leukemias Per Million

World Region	Country	Province/Region	Ethnic Group	Age range years	Period study	ASIR AL	ASIR ALL	ASIR AML	Authors
North America	USA	CDC	American	0–19	2001-2014	-	34	-	(Siegel et al. 2017)
North America	USA	Nationwide	Non-Hispanic White American	0–19	1990–2012	43.5	33.7	7.7	(Giddings et al. 2016)
North America	USA	CDC	Asian/Pacific Islander American	0–19	2001–2014	-	31.6	-	(Siegel et al. 2017)
North America	USA	Nationwide	Asian/Pacific Islander American	0–19	1990–2012	42.4	30.6	8.8	(Giddings et al. 2016)
North America	USA	CDC	American Indian/Alaskan/ Native American	0–19	2001–2014	-	30.2	-	(Siegel et al. 2017)
North America	USA	SEER	Non-Hispanic Asian American	0–19	1992–2013	42.1	29.7	10.4	(Barrington-Trimis et al. 2017)
North America	USA	Nationwide	Filipino American	0–19	1998–2002	36.4	27.6	7.2	(Moore et al. 2020)

ASIR, age-standardized incidence rates; AL, acute leukemias; ALL, acute lymphoblastic leukemia; AML, acute myeloblastic leukemia. *Crude Incidence Rates

Cont. SUPPLEMENTARY TABLE 1. Comparison of Age-Standardized Incidence Rates of All Childhood Acute Leukemias Per Million

World Region	Country	Province/Region	Ethnic Group	Age range years	Period study	ASIR AL	ASIR ALL	ASIR AML	Authors
North America	USA	CDC	African American	0–19	2001–2014	-	18.7	-	(Siegel et al. 2017)
North America	USA	Nationwide	African American	0–19	1990–2012	28.5	18.5	7.7	(Giddings et al. 2016)
North America	USA	Nationwide	Southeast Asian (Vietnamese, Laotian, Cambodian) American	0–19	1998–2002 and 1993–1997	29.4	22.2	6.6	(Moore et al. 2020)
North America	USA	SEER	Non-Hispanic Black American	0–19	1992–2013	26.2	18.3	6.7	(Barrington-Trimis et al. 2017)
Europe	Austria	Nationwide	Austrian	0–19	2009–2011	54.1	43.4	7.1	(Karim-Kos et al. 2016)
Europe	Italy	Piedmont	Italian	0–19	1967–2011	51.2	37.3	7.1	(Isaevska et al. 2017)
Asia	China	Henan	Chinese	0–19	2010–2014	42.5	8.6	3.6	(Q. Chen et al. 2019)

ASIR, age-standardized incidence rates; AL, acute leukemias; ALL, acute lymphoblastic leukemia; AML, acute myeloblastic leukemia. *Crude Incidence Rates

Cont. SUPPLEMENTARY TABLE 1. Comparison of Age-Standardized Incidence Rates of All Childhood Acute Leukemias Per Million

World Region	Country	Province/Region	Ethnic Group	Age range years	Period study	ASIR AL	ASIR ALL	ASIR AML	Authors
South America, Central America, and the Caribbean	Costa Rica	Nationwide	Costa Rican	0–19	2000–2014	58.5	46.9	6.7	(Erdmann et al. 2018)
South America, Central America, and the Caribbean	Brazil	Goiania-Goias	Brazilian	0–19	1996–2012	40.4	-	-	(Oliveira Friestino et al. 2018)
South America, Central America, and the Caribbean	Brazil	Pernambuco	Brazilian	0–19	2009–2012	34.8	25.1	8.3	(Bastos et al. 2018)

ASIR, age-standardized incidence rates; AL, acute leukemias; ALL, acute lymphoblastic leukemia; AML, acute myeloblastic leukemia. *Crude Incidence Rates

Note: The authors of the present study conducted a review of the literature reported in the last 6 years from 2015 to 2020 using PUBMED as the source. The keywords used were: childhood, leukemia, and incidence. All those studies that did not report incidence rates, but frequencies in percentages were not included; likewise, those investigations whose study methodology yielded outlier results. Only articles in English, Spanish and Portuguese were selected. The results were organized in the following order: 1) world region; 2) incidence rate; 3) age groups (0 to 14 and 0-19); and 4) ethnic group.

Bibliography

1. Abood, Rafid A., Kareem A. Abdahmed, and Seena S. Mazyed. 2020. "Epidemiology of Different Types of Cancers Reported in Basra, Iraq." *Sultan Qaboos University Medical Journal* 20 (3): 295–300. https://doi.org/10.18295/squmj.2020.20.03.008.
2. AIRTUM Working Group, CCM, and AIEOP Working Group. 2016. "I Tumori in Italia - Rapporto 2012 I Tumori Dei Bambini e Degli Adolescenti." *Epidemiologia e Prevenzione* 40 (1): 1–24.
3. Akhtar, Saeed, Jarrah Al-Abkal, and Ahmad Al-Shammari. 2020. "Childhood Leukaemia Incidence and Trends in a Middle Eastern Country during 1980–2014: A Population-Based Study." *Cancer Causes and Control* 31 (3): 231–40. https://doi.org/10.1007/s10552-020-01267-3.
4. Al-Asadi, Jasim N, and Sarah J Ibrahim. 2018. "Childhood Cancer in Basrah, Iraq During 2012-2016: Incidence and Mortality." *Asian Pacific Journal of Cancer Prevention* 19 (8): 2337–41.
5. Baade, P. D., D. R. Youlden, P. C. Valery, T. Hassall, L. Ward, A. C. Green, and J. F. Aitken. 2010. "Trends in Incidence of Childhood Cancer in Australia, 1983-2006." *British Journal of Cancer* 102 (3): 620–26. https://doi.org/10.1038/sj.bjc.6605503.
6. Barrington-Trimis, Jessica L., Myles Cockburn, Catherine Metayer, W. James Gauderman, Joseph Wiemels, and Roberta McKean-Cowdin. 2017. "Trends in Childhood Leukemia Incidence over Two Decades from 1992 to 2013." *International Journal of Cancer* 140 (5): 1000–1008. https://doi.org/10.1002/ijc.30487.
7. Bastos, Lídia N.V., José C Silveira, Carlos F Luna, and Norma Lucena-Silva. 2018. "Childhood and Adolescent Cancer in the State of Pernambuco, Brazil: Incidence, Geographical Distribution, and Association with Environmental Health Indicators." *Journal of Pediatric Hematology/Oncology* 40 (1): 7–14. https://doi.org/10.1097/MPH.0000000000001017.
8. Belgaumi, Asim F., Ghulam Q. Pathan, Khawar Siddiqui, Afshan A. Ali, Ibrahim Al-Fawaz, Suleimman Al-Sweedan, Mouhab Ayas, and Amani A. Al-Kofide. 2019. "Incidence, Clinical Distribution, and Patient Characteristics of Childhood Cancer in Saudi Arabia: A Population-Based Analysis." *Pediatric Blood and Cancer* 66 (6): e27684. https://doi.org/10.1002/pbc.27684.
9. Broccia, Giorgio, Jonathan Carter, Cansu Ozsin-Ozler, Federico Meloni, Ilaria Pilia, Giannina Satta, Giulio Murgia, Marcello Campagna, and Pierluigi Cocco. 2020. "Haemolymphatic Cancer among Children in Sardinia, Italy: 1974-2003 Incidence." *BMJ Open* 10 (11): 37163. https://doi.org/10.1136/bmjopen-2020-037163.
10. Chen, Qiong, Zhen Guo, Shuzheng Liu, Peiliang Quan, Xiaoqin Cao, Lanwei Guo, Shaokai Zhang, and Xibin Sun. 2019. "The Cancer Incidence and Mortality among Children and Adolescents during the Period of 2010-2014 in Henan Province, China." *Cancer Medicine* 8 (2): 814–23. https://doi.org/10.1002/cam4.1952.
11. Chen, Xuanwei, Jianwei Pan, Shuncong Wang, Shandie Hong, Shunrong Hong, and Shaoru He. 2019. "The Epidemiological Trend of Acute Myeloid Leukemia in Childhood: A Population-Based Analysis." *Journal of Cancer* 10 (20): 4824–35. https://doi.org/10.7150/jca.32326.
12. Erdmann, Friederike, Tengfei Li, George Luta, Brenda M. Giddings, Guillermo Torres Alvarado, Eva Steliarova-Foucher,

Joachim Schüz, and Ana M. Mora. 2018. “Incidence of Childhood Cancer in Costa Rica, 2000–2014: An International Perspective.” *Cancer Epidemiology* 56 (October): 21–30. https://doi.org/10.1016/j.canep.2018.07.004.

13. Fajardo-Gutiérrez, Arturo, Guadalupe González-Miranda, Adriana Pachuca-Vázquez, Aldo Allende-López, Liria Mitzuko Fajardo-Yamamoto, and Mario Enrique Rendón-Macías. 2016. “Cancer Incidence and Mortality in Children in the Mexican Social Security Institute (1996-2013).” *Salud Publica de Mexico* 58 (2): 162–70. https://doi.org/10.21149/spm.v58i2.7785.
14. Fajardo-Gutiérrez, Arturo, Servando Juárez-Ocaña, Guadalupe González-Miranda, Virginia Palma-Padilla, Rogelio Carreón-Cruz, Manuel Carlos Ortega-Alvárez, and Juan Manuel Mejía-Arangure. 2007. “Incidence of Cancer in Children Residing in Ten Jurisdictions of the Mexican Republic: Importance of the Cancer Registry (a Population-Based Study).” *BMC Cancer* 7 (January): 68.
15. Giddings, Brenda M., Todd P. Whitehead, Catherine Metayer, and Mark D. Miller. 2016. “Childhood Leukemia Incidence in California: High and Rising in the Hispanic Population.” *Cancer* 122 (18): 2867–75. https://doi.org/10.1002/cncr.30129.
16. Glazer, E R, C I Perkins, J L Young, R D Schlag, S L Campleman, and W E Wright. 1999. “Cancer among Hispanic Children in California, 1988-1994: Comparison with Non-Hispanic White Children.” *Cancer* 86 (6): 1070–79.
17. González-García, Hermenegildo, Rebeca Garrote-Molpeceres, Elena Urbaneja-Rodríguez, Pilar Gutiérrez-Meléndez, Raquel Herráiz-Cristóbal, María Asunción Pino-Vázquez, Hermenegildo González García, et al. 2018. “Differences in Incidence and Survival to Childhood Cancer between Rural and Urban Areas in Castilla y León, Spain (2003–2014).” *Medicine* 97 (41): e12797. https://doi.org/10.1097/MD.0000000000012797.
18. Holland, Lucy R., Natalie K. Bradford, Philippa Youl, Danica Cossio, Nathan Dunn, Nancy Tran, and Rick Walker. 2020. “Cancer Incidence, Mortality, and Survival for Children, Adolescents, and Young Adults in Queensland Between 1987 and 2016.” *Journal of Adolescent and Young Adult Oncology* December 2 (December): 16. https://doi.org/10.1089/jayao.2020.0151.
19. Isaevska, Elena, Milena Manasievska, Daniela Alessi, Maria Luisa Mosso, Corrado Magnani, Carlotta Sacerdote, Guido Pastore, Franca Fagioli, Franco Merletti, and Milena Maule. 2017. “Cancer Incidence Rates and Trends among Children and Adolescents in Piedmont, 1967–2011.” Edited by Maria Fiammetta Romano. *PLOS ONE* 12 (7): e0181805. https://doi.org/10.1371/journal.pone.0181805.
20. Jakab, Zsuzsanna, Attila Juhasz, Csilla Nagy, Dezso Schuler, Miklos Garami, and Hungarian Paediatric Haemato-Oncology Network. 2017. “Trends and Territorial Inequalities of Incidence and Survival of Childhood Leukaemia and Their Relations to Socioeconomic Status in Hungary, 1971-2015.” *European Journal of Cancer Prevention* 26 (September): S183–90. https://doi.org/10.1097/CEJ.0000000000000386.
21. Jastaniah, Wasil, Mohammed F. Essa, Walid Ballourah, Ibrahim Abosoudah, Saad Al Daama, Ali H. Algiraigri, Ibrahim Al Ghemlas, Mohammad Alshahrani, and Abdulrahman Alsultan. 2020. “Incidence Trends of Childhood Acute Lymphoblastic Leukemia in Saudi Arabia: Increasing Incidence or Competing Risks?” *Cancer Epidemiology* 67 (August): 101764.

https://doi.org/10.1016/j.canep.2020.101764.

22. Karim-Kos, Henrike E., Monika Hackl, Georg Mann, Christian Urban, Adelheid Woehrer, Irene Slavc, and Ruth Ladenstein. 2016. “Trends in Incidence, Survival and Mortality of Childhood and Adolescent Cancer in Austria, 1994-2011.” *Cancer Epidemiology* 42 (June): 72–81. https://doi.org/10.1016/j.canep.2016.03.015.
23. Krejci, Denisa, Michaela Zapletalova, Ivana Svobodova, Viera Bajciova, Peter Mudry, Vratislav Smelhaus, Jaroslav Sterba, Jan Stary, Riccardo Capocaccia, and Ladislav Dusek. 2020. “Childhood Cancer Epidemiology in the Czech Republic (1994–2016).” *Cancer Epidemiology* 69 (December): 101848. https://doi.org/10.1016/j.canep.2020.101848.
24. Mejía-Aranguré, Juan Manuel, Miguel Bonilla, Rodolpho Lorenzana, Servando Juárez-Ocaña, Gladys de Reyes, María Luisa Pérez-Saldivar, Guadalupe González-Miranda, et al. 2005. “Incidence of Leukemias in Children from El Salvador and Mexico City between 1996 and 2000: Population-Based Data.” *BMC Cancer* 5 (January): 33. https://doi.org/10.1186/1471-2407-5-33.
25. Mejía-Aranguré, Juan Manuel, Juan Carlos Núñez-Enríquez, Arturo Fajardo-Gutiérrez, María Del Carmen Rodríguez-Zepeda, Jorge Alfonso Martín-Trejo, David Aldebarán Duarte-Rodríguez, Aurora Medina-Sansón, et al. 2016. “Epidemiología Descriptiva de La Leucemia Mieloide Aguda (LMA) En Niños Residentes de La Ciudad de México: Reporte Del Grupo Mexicano Interinstitucional Para La Identificación de Las Causas de La Leucemia En Niños.” *Gaceta Médica de Mexico* 152 (Suppl 2): 66–77.
26. Moore, Kristin J., Aubrey K. Hubbard, Lindsay A. Williams, and Logan G. Spector. 2020. “Childhood Cancer Incidence among Specific Asian and Pacific Islander Populations in the United States.” *International Journal of Cancer* 147 (12): 3339–48. https://doi.org/10.1002/ijc.33153.
27. Moreno, Florencia, Dora Loria, Graciela Abriata, and Benedetto Terracini. 2013. “Childhood Cancer: Incidence and Early Deaths in Argentina, 2000-2008.” *European Journal of Cancer* 49 (2): 465–73. https://doi.org/10.1016/j.ejca.2012.08.001.
28. Oliveira Friestino, Jane Kelly, Denisa Mendonça, Pedro Oliveira, Carla M Oliveira, and Djalma de Carvalho Moreira Filho. 2018. “Cáncer Infantil: Incidencia y Patrones Espaciales En La Ciudad de Campinas, Brasil, 1996-2005.” *Salud Colectiva* 14 (1): 51–63. https://doi.org/https://doi.org/10.18294/sc.2018.1200.
29. Otoo, Marianne N., Martie S. Lubbe, Hanlie Steyn, and Johanita R. Burger. 2020. “Childhood Cancers in a Section of the South African Private Health Sector: Analysis of Medicines Claims Data.” *Health SA Gesondheid* 25 (September). https://doi.org/10.4102/hsag.v25i0.1382.
30. Paapsi, Keiu, Aleksei Baburin, Sirje Mikkel, Margit Mägi, Kadri Saks, and Kaire Innos. 2020. “Childhood Cancer Incidence and Survival Trends in Estonia (1970-2016): A Nationwide Population-Based Study.” *BMC Cancer* 20 (1): 30. https://doi.org/10.1186/s12885-019-6510-7.
31. Pérez-Saldivar, María Luisa, Arturo Fajardo-Gutiérrez, Roberto Bernáldez-Ríos, Armando Martínez-Avalos, Aurora Medina-Sanson, Laura Espinosa-Hernández, José de Diego Flores-Chapa, et al. 2011. “Childhood Acute Leukemias Are Frequent in Mexico City: Descriptive Epidemiology.” *BMC Cancer* 11 (1): 355. https://doi.org/10.1186/1471-2407-11-355.

32. Peris-Bonet, R., D. Salmerón, M. A. Martínez-Beneito, J. Galceran, R. Marcos-Gragera, S. Felipe, V. González, et al. 2010. "Childhood Cancer Incidence and Survival in Spain." *Annals of Oncology* 21 (SUPPL.3). https://doi.org/10.1093/annonc/mdq092.
33. Rahimi Pordanjani, Sajjad, Amir Kavousi, Babak Mirbagheri, Abbas Shahsavani, and Koorosh Etemad. 2020. "Temporal Trend and Spatial Distribution of Acute Lymphoblastic Leukemia in Iranian Children during 2006-2014: A Mixed Ecological Study." *Epidemiology and Health* 42: e2020057. https://doi.org/10.4178/epih.e2020057.
34. Reedijk, Ardine M.J., Leontien C. Kremer, Otto Visser, Valery Lemmens, Rob Pieters, Jan Willem W. Coebergh, and Henrike E. Karim-Kos. 2020. "Increasing Incidence of Cancer and Stage Migration towards Advanced Disease in Children and Young Adolescents in the Netherlands, 1990–2017." *European Journal of Cancer* 134 (July): 115–26. https://doi.org/10.1016/j.ejca.2020.04.011.
35. Services, Texas Department of State Health. 2020. "Texas Cancer Registry. SEER*Stat Database, 1995-2017 Incidence, Texas Statewide." Childhood and Adolescent Cancer. January 2020. https://www.dshs.state.tx.us/tcr/childhood.shtm.
36. Shabani, Mahsima, Sahar Saeedi Moghaddam, Bahar Ataeinia, Nazila Rezaei, Farnam Mohebi, Bahram Mohajer, Kimiya Gohari, et al. 2020. "Trends of National and Subnational Incidence of Childhood Cancer Groups in Iran: 1990–2016." *Frontiers in Oncology* 9 (January): 1428. https://doi.org/10.3389/fonc.2019.01428.
37. Siegel, David A., S. Jane Henley, Jun Li, Lori A. Pollack, Elizabeth A. Van Dyne, and Arica White. 2017. "Rates and Trends of Pediatric Acute Lymphoblastic Leukemia — United States, 2001–2014." *MMWR. Morbidity and Mortality Weekly Report* 66 (36): 950–54. https://doi.org/10.15585/mmwr.mm6636a3.
38. Sommer, Grit, Matthias Schindler, Shelagh Redmond, Verena Pfeiffer, Garyfallos Konstantinoudis, Roland A. Ammann, Marc Ansari, H. Hengartner, Gisela Michel, and Claudia E. Kuehni. 2019. "Temporal Trends in Incidence of Childhood Cancer in Switzerland, 1985–2014." *Cancer Epidemiology* 61 (August): 157–64. https://doi.org/10.1016/j.canep.2019.06.002.
39. Stefan, Cristina, Freddie Bray, Jacques Ferlay, Biying Liu, and D. Maxwell Parkin. 2017. "Cancer of Childhood in Sub-Saharan Africa." *Ecancermedicalscience*. Cancer Intelligence. https://doi.org/10.3332/ecancer.2017.755.
40. Steliarova-Foucher, E, M Colombet, LAG Ries, P Hesseling, F Moreno, HY Shin, and CA Stiller. 2017. "International Incidence of Childhood Cancer, Volume III (Electronic Version)." Edited by E Steliarova-Foucher, M Colombet, LAG Ries, P Hesseling, F Moreno, HY Shin, and CA Stiller. Lyon, France: International Agency for Research on Cancer. 2017. http://iicc.iarc.fr/results/citation.php.
41. Wiangnon, Surapon, Arunee Jetsrisuparb, Patcharee Komvilaisak, and Krittika Suwanrungruang. 2014. "Childhood Cancer Incidence and Survival 1985-2009, Khon Kaen, Thailand." *Asian Pacific Journal of Cancer Prevention* 15 (18): 7989–93. https://doi.org/10.7314/APJCP.2014.15.18.7989.
42. Wilkinson, James D., Lora E. Fleming, Jill MacKinnon, Lydia Voti, Bradley Wohler-Torres, Steven Peace, and Edward Trapido. 2001. "Lymphoma and Lymphoid Leukemia Incidence in Florida Children." *Cancer* 91 (7): 1402–8.

https://doi.org/10.1002/1097-0142(20010401)91:7<1402::AID-CNCR1145>3.0.CO;2-6.

43. Youlden, Danny R, Peter D Baade, Adèle C Green, Patricia C Valery, Andrew S Moore, and Joanne F Aitken. 2020. “The Incidence of Childhood Cancer in Australia, 1983–2015, and Projections to 2035.” *Medical Journal of Australia* 212 (3): 113–20. https://doi.org/10.5694/mja2.50456.
